# Comorbid Depression Among US Adults With Elastography-Defined Advanced Chronic Liver Disease and Clinically Significant Portal Hypertension: National Health and Nutrition Examination Survey 2017–2023

**DOI:** 10.1016/j.gastha.2026.101112

**Published:** 2026-09-03

**Authors:** Sarpong Boateng, Guy Loic Nguefang, Mexan Mapouka, Bright Nwatamole, Solomon Gyabaah, Henry Onyeaka, Leandro Sierra, Yazan Al-Aljouni, Basile Njei

**Affiliations:** 1 Division of Digestive Diseases, Yale University, New Haven, Connecticut; 2 Department of Medicine, Yale New Haven Health, Bridgeport Hospital, Bridgeport, Connecticut; 3 Department of Medicine, Texas Tech University Health Sciences Center, Lubbock, Texas; 4 Department of Medicine, University of Maryland Capital Region Medical Center, Largo, Maryland; 5 Department of Medicine, Zucker School of Medicine at Hofstra/ Northwell at Vassar Brothers Medical Center, Poughkeepsie, New York; 6 Department of Medicine, Komfo Anokye Teaching Hospital, Kumasi, Ghana; 7 Department of Psychiatry, Massachusetts General Hospital, Boston, Massachusetts; 8 Department of Medicine, Cleveland Clinic Foundation, Cleveland, Ohio; 9 Department of Rehabilitation, Montefiore Medical Center, Bronx, New York

**Keywords:** Vibration-Controlled Transient Elastography, Cirrhosis, Mental Health, Patient Health Questionnaire-9, COVID-19

## Abstract

**Background and Aims:**

Depression is a critical yet underrecognized comorbidity in chronic liver disease, with important implications for treatment adherence, self-management, and clinical outcomes. However, nationally representative estimates of comorbid depression using contemporary elastography-defined advanced chronic liver disease (ACLD) and clinically significant portal hypertension (CSPH) are lacking.

**Methods:**

We analyzed adults from the National Health and Nutrition Examination Survey 2017–2020 and 2021–2023 cycles using valid vibration-controlled transient elastography, Patient Health Questionnaire-9 data, and complete covariates. Survey-weighted logistic regression identified factors associated with depression in ACLD patients, adjusting for the identified covariates. Sensitivity analyses were used to evaluate alternative Patient Health Questionnaire-9 scoring assumptions.

**Results:**

Among 13,291 participants representing the US adult population, the weighted prevalence of comorbid depression was 12.16% with 95% confidence interval (CI) 8.65, 15.66%. In multivariate analysis, female sex (adjusted odds ratio [aOR] 3.51; 95% CI 1.98, 6.23; *P* < .001) and greater sedentary time (aOR 1.012 per hour/day; 95% CI 1.006, 1.018; *P* = .021) were independently associated with higher odds of comorbid depression. Older age (aOR 0.97 per year; 95% CI 0.94, 0.99; *P* = .025) and non-Hispanic Black race compared with non-Hispanic White race (aOR 0.27; 95% CI 0.10, 0.71; *P* = .010) were associated with lower odds of depression in ACLD. The odds of depression were 78% higher in the postpandemic period than in the prepandemic (aOR 1.78; 95% CI 1.04, 3.02; *P* = .035). The findings were robust across sensitivity analyses.

**Conclusion:**

Approximately 12% to 13% of US adults with ACLD or CSPH experience comorbid depression, with an increase in the post–COVID-19 era. Depression burden was the highest among women, younger adults, individuals with high sedentary time, and non-Hispanic Black adults with CSPH. Therefore, there is a need to integrate behavioral health pathways into hepatology care.

## Introduction

The United States has experienced an increased prevalence of depression during the recent COVID-19 pandemic, compounding the burden of pre-existing chronic conditions, such as obesity, chronic liver disease (CLD), and cardiovascular disease.^1^ The COVID-19 pandemic has accelerated this burden by exacerbating metabolic risk factors through increased sedentary behavior, unhealthy diet, and obesity.^1^ CLD has long been recognized and associated with depression.^2^ Individuals living with CLDs often experience dysregulation of hepatic inflammatory pathways. Persistent hepatic inflammation leads to elevated circulating cytokines, impaired detoxification of neurotoxic substances, and dysregulation of neurotransmitter systems and mood.^3^ In addition, the financial and social burden associated with CLD is not negligible and may further disrupt mood regulation, contributing to depression and other mental health conditions.^4^ Individuals facing socioeconomic barriers in the United States, including disadvantaged racial and ethnic minority groups with metabolic dysfunction–associated steatotic liver disease (MASLD), often experience coexisting obesity and adverse social health determinants. Those with low incomes, housing instability, and limited access to transportation are not spared.^5^ These factors are independently and collectively associated with poorer mental health outcomes and impose additional stress compared with the general population.

Psychiatric conditions, including depression, may also worsen CLD care by limiting access to care, reducing adherence to medical and lifestyle recommendations, and impairing follow-up with hepatology services. Prior studies have shown that depression and anxiety in CLD are associated with reduced compliance and poorer clinical outcomes, while financial burden may further disrupt longitudinal care.^4^^,^^6^ Greater medical comorbidity and socioeconomic deprivation may also adversely affect health-related quality of life, which may in turn contribute to depression and anxiety in liver disease populations.^7^ Despite these observations, the relationship between depression and CLD remains insufficiently studied in the existing literature. Whether there is a positive correlation between the development of depression and specific chronic liver conditions remains unclear.

This study aimed to explore the relationship between CLDs and depression, with a particular focus on identifying risk factors that increase the likelihood of depression among individuals living with CLD.

## Methods

### Study Design and Data Source

We conducted a cross-sectional analysis of the National Health and Nutrition Examination Survey (NHANES), a nationally representative survey of the noninstitutionalized US population conducted by the National Center for Health Statistics, using a complex, multistage probability sampling design. We combined data from the 2017 to 2020 and 2021 to 2023 NHANES cycles in accordance with the Centers for Disease Control and Prevention guidance for pooled analyses across survey cycles, applying appropriately adjusted examination weights to generate nationally representative estimates.

The NHANES includes standardized interviews, physical examinations, laboratory testing, and vibration-controlled transient elastography (VCTE), enabling contemporary noninvasive assessment of liver disease severity alongside validated mental health screening instruments. All NHANES participants provided written informed consent and the survey protocol was approved by the National Center for Health Statistics Research Ethics Review Board. Because this study used deidentified publicly available data, it was exempt from the institutional review board review.

### Study Population

We included adults aged ≥20 years with valid VCTE liver stiffness measurements (LSMs) and available data on depression screening and covariates of interest. Participants were excluded if VCTE data were missing or unreliable, consistent with NHANES quality assurance protocols. For analyses involving depression severity, participants were included based on the specific Patient Health Questionnaire-9 (PHQ-9) scoring strategy applied, as described below.

### Definitions of Advanced Chronic Liver Disease and Clinically Significant Portal Hypertension

#### Advanced chronic liver disease

Advanced chronic liver disease (ACLD) was defined using LSM obtained by VCTE. In the primary analysis, ACLD was defined as LSM >12 kPa, consistent with prior population-based studies and contemporary noninvasive frameworks aligned with the American Association for the Study of Liver Diseases guidance.

Sensitivity analyses were further examined as follows:

• LSM 10 to 15 kPa (suggestive of ACLD), and
• LSM >15 kPa (highly suggestive of ACLD).

#### Clinically significant portal hypertension

Clinically significant portal hypertension (CSPH) was defined using validated elastography-based criteria incorporating platelet count, as follows:

• LSM 15 to 20 kPa with platelet count <110 × 10^9^/L,
• LSM >20 to 25 kPa with platelet count <150 × 10^9^/L, or
• LSM >25 kPa regardless of platelet count.

These thresholds reflect the established noninvasive definitions of CSPH used in both clinical and epidemiological studies.

#### Definition of excessive alcohol consumption

Excessive alcohol consumption was defined using NHANES alcohol-use questionnaire data based on standard sex-specific thresholds. For men, excessive consumption was defined as >14 drinks per week, and for women, >7 drinks per week, consistent with established public health guidelines. This definition reflects alcohol intake above recommended limits and does not correspond to a clinical diagnosis of alcohol use disorder. Alcohol use was incorporated into liver disease etiology classification as alcohol-associated liver disease where applicable.

### Assessment of Depression

#### Patient Health Questionnaire-9 instrument

Depression was assessed using the PHQ-9, a validated screening tool for depressive symptoms that is widely used in population-based studies and clinical practice. The PHQ-9 evaluates the frequency of 9 depressive symptoms over the previous 2 weeks, with each item scored from 0 to 3, yielding a total score ranging from 0 to 27.

#### Primary definition of comorbid depression

For the primary analysis, comorbid depression was defined as a PHQ-9 score ≥10, a threshold with established sensitivity and specificity for clinically significant depressive disorders. Severe depression was defined as PHQ-9 ≥15.

#### Depression severity categories

PHQ-9 scores were categorized into standard severity groups as follows:

• Minimal: 0 to 4
• Mild: 5 to 9
• Moderate: 10 to 14
• Moderately severe: 15 to 19
• Severe: 20 to 27

#### Handling of missing Patient Health Questionnaire-9 data and sensitivity analyses

Depression scores were reconstructed directly from the individual PHQ-9 items (DPQ010–DPQ090). The following 3 complementary analytical strategies were used:

1 Primary (complete-case) analysis: PHQ-9 scores were calculated only for participants with all 9 PHQ-9 items completed. Participants with missing PHQ-9 items were excluded from depression analyses using this approach.
2 Sensitivity analysis 1: ≥7 items present with mean imputation: PHQ-9 scores were calculated for participants with at least 7 completed items. Missing items were imputed using the mean of available responses, consistent with validated PHQ-9 scoring practices in epidemiological research.
3 Sensitivity analysis 2: missing treated as zero: All missing PHQ-9 items were assigned a score of zero, representing a conservative assumption of no depressive symptoms. This approach mirrors the methods used in prior NHANES-based depression studies and provides a lower-bound estimate of the prevalence of depression.

Results across these approaches were compared to assess robustness of prevalence estimates and associations.

#### Covariates

Covariates were selected a priori based on clinical relevance and prior literature and included the following:

• Demographics: age (continuous), sex, race/ethnicity (Mexican American, other Hispanic, non-Hispanic White, non-Hispanic Black, non-Hispanic Asian, non-Hispanic other).
• Socioeconomic factors: education level (less than high school, high school or equivalent, above high school), marital status (married/living with partner, widowed/divorced/separated, never married), and poverty-income ratio (<1.30, 1.30–3.49, ≥3.50)
• Lifestyle factors: smoking status (current, past, nonsmoker), sedentary activity (hours/day, modeled continuously and categorically), and alcohol use. Excessive alcohol consumption was derived from NHANES alcohol-use questionnaire variables and was used to classify alcohol-associated liver disease etiology. Other substance use disorders were not included because harmonized measures were not consistently available across the included NHANES cycles.
• Clinical factors: body mass index (BMI; <25.0, 25.0–29.9, ≥30.0 kg/m^2^), diabetes, hypertension, hyperlipidemia
• Liver-specific variables: self-reported history of liver disease, current liver disease, and etiology of liver disease (MASLD, metabolic dysfunction and alcohol-associated liver disease, excessive alcohol consumption/alcohol-associated liver disease, viral hepatitis B/C, or other). Alcohol-associated liver disease was classified using the NHANES alcohol-use questionnaire variables in combination with the liver disease etiology algorithm.

The survey era was categorized as pre–COVID-19 (2017–March 2020) or COVID-19 (August 2021–August 2023).

### Statistical Analysis

All analyses incorporated NHANES survey design variables, including strata, primary sampling units, and examination weights, to account for the complex sampling design and to produce nationally representative estimates.

Weighted prevalence estimates with 95% confidence intervals (CIs) were calculated for comorbid depression among individuals with ACLD and CSPH and compared with estimates from the general US adult population. Prevalence estimates were stratified according to demographic, socioeconomic, lifestyle, and clinical characteristics.

Multivariable logistic regression was used to identify factors independently associated with comorbid depression in adults with ACLD. The models were adjusted for age, sex, race/ethnicity, marital status, education, poverty-income ratio, smoking status, sedentary activity, BMI, metabolic comorbidities, liver disease etiology, self-reported liver disease, and survey era. Adjusted odds ratios (aORs) with 95% CIs are reported.

All analyses were conducted using R (version 4.5.1) and SAS (version 9.4) with survey-specific packages. Statistical significance was defined as a two-sided *P* value of <.05.

## Results

### Study Population

The analytic cohort comprised 13,291 adults from NHANES 2017 to 2023 with valid VCTE measurements and depression assessments, representing the noninstitutionalized US adult population after the application of survey weights. Among these participants, 96 individuals met criteria for comorbid depression in elastography-defined ACLD and 23 met criteria for CSPH, corresponding to approximately 1.51 million adults with comorbid depression with ACLD and 323,000 adults with comorbid depression and CSPH nationwide (Table 1).

**Table 1:** Weighted Prevalence of Comorbid Depression and ACLD/CSPH in the US Population 2017–2023

Population	ACLD^a^		CSPH^b^		US adult population comparison	
	Comorbid depression (PHQ-9 ≥10)		Comorbid depression (PHQ-9 ≥10)		Depression (PHQ-9 ≥10)	
	N (weighted N)	% of all cases	N (weighted N)	% of all cases	N (weighted N)	% of all cases
All persons	96 (1,510,162)	12.16 (8.65, 15.66)	23 (323,422)	12.75 (5.81, 19.68)	1304 (21,648,287)	9.56 (8.67, 10.45)
Age group						
18–40 y	25 (480,152)	16.78 (7.34, 26.22)	5 (71,510)	18.44 (0.00, 37.58)	534 (10,781,384)	11.99 (10.28, 13.70)
41–60 y	34 (562,332)	11.46 (7.98, 14.94)	8 (120,413)	10.51 (3.55, 17.48)	417 (6,714,331)	8.90 (8.00, 9.80)
61–80 y	37 (467,678)	10.05 (5.51, 14.59)	10 (131,499)	13.09 (3.77, 22.42)	353 (4,152,572)	6.80 (5.83, 7.77)
Sex						
Male	34 (531,966)	7.03 (4.32, 9.73)	10 (150,067)	8.12 (1.03, 15.22)	487 (8,475,770)	7.62 (6.58, 8.65)
Female	62 (978,196)	20.16 (13.53, 26.78)	13 (173,355)	25.15 (9.54, 40.76)	817 (13,172,517)	11.44 (10.19, 12.70)
Race/ethnicity						
Mexican American	8 (123,754)	11.89 (2.51, 21.27)	1 (7671)	4.57 (0.00, 14.73)	128 (1,707,889)	9.58 (6.93, 12.23)
Other Hispanic	8 (116,001)	14.29 (4.24, 24.33)	1 (26,266)	11.16 (0.00, 33.54)	148 (1,943,140)	9.93 (8.24, 11.62)
Non-Hispanic White	60 (1,016,949)	12.02 (8.24, 15.80)	13 (220,361)	12.19 (3.04, 21.35)	620 (13,153,069)	9.43 (8.36, 10.50)
Non-Hispanic Black	9 (58,128)	5.98 (1.78, 10.18)	5 (39,183)	26.88 (7.94, 45.82)	240 (2,497,567)	10.12 (7.74, 12.49)
Non-Hispanic Asian	4 (44,574)	15.68 (0.00, 36.58)	1 (4769)	21.96 (0.00, 47.26)	48 (576,729)	4.42 (2.72, 6.12)
Non-Hispanic other	7 (150,756)	17.65 (0.50, 34.80)	2 (25,173)	15.80 (0.00, 39.28)	120 (1,769,893)	14.92 (11.52, 18.33)
Marital status						
Married/living with partner	11 (132,424)	5.94 (2.21, 9.68)	5 (61,428)	11.16 (2.47, 19.85)	242 (3,618,445)	5.91 (5.17, 6.64)
Widowed/divorced/separated	35 (486,065)	17.67 (9.86, 25.47)	8 (110,329)	18.41 (7.11, 29.72)	374 (5,056,241)	12.59 (11.19, 14.00)
Never married	22 (402,744)	18.45 (10.00, 26.90)	5 (85,392)	18.83 (0.00, 38.20)	351 (6,257,804)	14.09 (11.96, 16.22)
Level of education						
Less than high school	18 (200,782)	12.46 (6.60, 18.31)	3 (37,526)	10.13 (0.00, 23.21)	223 (2,529,760)	11.53 (9.27, 13.80)
High school or equivalent	25 (479,405)	11.23 (5.12, 17.35)	9 (182,478)	21.31 (0.00, 43.32)	330 (6,377,565)	10.95 (9.62, 12.28)
Above high school	47 (758,654)	11.83 (7.78, 15.88)	9 (91,996)	7.10 (2.80, 11.40)	658 (11,489,470)	8.27 (7.26, 9.29)
Poverty-income ratio						
<1.30	35 (576,213)	21.96 (13.53, 30.39)	7 (97,293)	18.91 (1.09, 36.74)	420 (5,998,390)	15.68 (13.66, 17.70)
1.30–3.49	35 (618,311)	13.66 (7.42, 19.90)	10 (134,184)	14.35 (0.00, 29.87)	486 (8,265,462)	11.56 (10.10, 13.02)
≥3.50	17 (205,305)	5.62 (1.67, 9.57)	4 (69,284)	11.57 (1.67, 21.46)	235 (5,068,229)	5.71 (4.79, 6.64)
Sedentary activity, hours/day						
<4	10 (96,015)	3.40 (1.10, 5.70)	1 (4214)	0.63 (0.00, 1.96)	318 (4,861,473)	8.27 (6.96, 9.59)
4–<6	25 (494,494)	15.88 (6.65, 25.11)	6 (60,478)	14.14 (0.02, 28.27)	300 (5,286,729)	9.25 (7.65, 10.86)
6–<8	12 (175,378)	9.61 (4.49, 14.73)	3 (50,095)	13.90 (0.00, 29.34)	220 (3,442,089)	9.77 (7.67, 11.87)
≥8	49 (744,275)	16.18 (10.73, 21.63)	13 (208,635)	19.79 (4.21, 35.38)	455 (7,955,228)	10.76 (9.38, 12.14)
BMI categories, Kg/m^2^						
<25	12 (148,448)	13.71 (1.64, 25.77)	3 (44,771)	33.06 (0.00, 66.18)	306 (5,942,971)	9.35 (7.76, 10.95)
25–29.9	13 (163,677)	8.11 (3.06, 13.15)	4 (48,017)	10.30 (0.00, 24.04)	361 (5,549,309)	7.71 (6.56, 8.86)
≥30	71 (1,198,036)	12.85 (8.96, 16.74)	16 (230,635)	11.92 (4.87, 18.96)	637 (10,156,007)	11.17 (9.87, 12.47)
Smoking status						
Current smoker	20 (248,804)	18.38 (11.37, 25.39)	5 (64,815)	30.20 (0.00, 66.32)	284 (4,617,194)	17.10 (14.62, 19.58)
Past smoker	25 (399,798)	10.55 (4.11, 16.99)	8 (135,388)	13.85 (4.32, 23.39)	296 (4,814,505)	8.93 (7.51, 10.34)
Nonsmoker	51 (861,560)	11.83 (8.04, 15.63)	10 (123,219)	9.16 (2.38, 15.94)	724 (12,216,587)	8.40 (7.53, 9.26)
Known self-reported liver condition ever						
Yes	27 (419,885)	24.19 (9.83, 38.56)	8 (105,554)	18.79 (1.05, 36.52)	120 (1,859,928)	17.58 (13.83, 21.34)
No	62 (1,015,138)	9.63 (6.49, 12.76)	13 (206,444)	10.59 (4.31, 16.86)	1086 (18,501,206)	8.89 (7.99, 9.78)
Known current liver condition						
Yes	22 (356,094)	24.60 (13.78, 35.42)	7 (101,340)	19.89 (0.00, 39.99)	79 (1,168,446)	19.40 (14.42, 24.39)
No	2 (22,801)	17.25 (0.00, 36.08)	0 (0)	-	31 (540,890)	16.15 (9.24, 23.07)
Etiology of liver disease, n and % of all cases						
MASLD	54 (911,580)	11.33 (7.65, 15.00)	16 (224,596)	13.95 (6.07, 21.82)	450 (7,240,548)	9.92 (8.90, 10.93)
MetALD	4 (43,917)	8.87 (0.00, 20.43)	1 (4769)	4.98 (0.31, 9.66)	20 (360,358)	8.98 (4.41, 13.55)
Excessive alcohol consumption	7 (19 × 9,090)	22.63 (2.18, 43.08)	2 (62,099)	53.42 (0.00, 100.00)	86 (1,804,291)	16.15 (11.46, 20.84)
Viral hepatitis (B and C)^c^	8 (62,513)	15.88 (0.00, 32.73)	2 (19,635)	19.09 (0.00, 45.76)	41 (517,526)	16.93 (10.88, 22.98)
None of the above	23 (293,062)	11.24 (6.92, 15.56)	2 (12,324)	2.01 (0.00, 5.20)	707 (11,725,563)	8.68 (7.72, 9.63)

MetALD, metabolic dysfunction and alcohol-associated liver disease

a LSM >12 kPa was used as a single threshold definition of ACLD, in accordance with prior studies.

b CSPH defined as LSM 15 to 20 kPa with PLT <110 G/L, or LSM >20 kPa and ≤25 kPa with PLT <150 G/L, or LSM >25 kPa regardless of platelet count.

c For the 2021–2023 cycle, viral hepatitis was estimated by self-reported diagnosis.

### Prevalence of Comorbid Depression in Advanced Chronic Liver Disease and Clinically Significant Portal Hypertension

Overall, the weighted prevalence of comorbid depression (PHQ-9 ≥10) was 12.16% (95% CI 8.65, 15.66) among adults with ACLD and 12.75% (95% CI 5.81, 19.68) among those with CSPH, compared with 9.56% (95% CI 8.67, 10.45) in the general US adult population (Table 1).

Across age strata, comorbid depression was the most prevalent among younger adults aged 18 to 40 years, affecting 16.78% of those with ACLD and 18.44% of those with CSPH, with a progressive decline observed in the older age groups. Women exhibited a substantially higher prevalence than men, with depression affecting 20.16% of women with ACLD and 25.15% of women with CSPH, compared with 7.03% and 8.12% among men, respectively.

Differences in the prevalence were observed according to race and ethnicity. Among individuals with ACLD, the prevalence of comorbid depression ranged from 5.98% in non-Hispanic Black adults to 17.65% in non-Hispanic other adults. In contrast, among those with CSPH, the prevalence of depression was notably elevated among non-Hispanic Black adults (26.88%) and non-Hispanic Asian adults (21.96%).

#### Socioeconomic, behavioral, and clinical correlates

Socioeconomic gradients in depression prevalence were pronounced. Adults with ACLD and a poverty-income ratio <1.30 had a depression prevalence of 21.96%, compared with 5.62% among those with a ratio ≥3.50. Similar patterns were observed among individuals with CSPH 18.91% and 11.57%, respectively.

Sedentary behavior demonstrated a dose–response relationship with depression prevalence. Among adults with ACLD, depression prevalence increased from 3.40% among those sedentary for <4 h/day to 16.18% among those sedentary for ≥8 h/day. A comparable gradient was observed among individuals with CSPH, with depression prevalence reaching 19.79% in the highest sedentary category.

Current smoking was associated with higher depression prevalence in both ACLD (18.38%) and CSPH (30.20%) compared with nonsmokers. Alcohol-related patterns were evaluated through the liver disease etiology classification, with excessive alcohol consumption analyzed separately as an etiologic category. Other substance use, including marijuana, opioid, and stimulant use, was not evaluated because harmonized measures were not consistently available across the included NHANES cycles. Obesity (BMI ≥30 kg/m^2^) was common among individuals with ACLD and CSPH, although depression prevalence did not differ substantially across BMI categories.

#### Depression prevalence by liver disease etiology and excessive alcohol consumption

The prevalence of depression varied according to liver disease etiology, including excessive alcohol consumption as defined in the Methods (Table 1). Among adults with ACLD, comorbid depression was the most prevalent in those with excessive alcohol consumption (22.63%), followed by viral hepatitis B or C (15.88%), and MASLD (11.33%). Individuals with CSPH and excessive alcohol consumption exhibited a particularly high prevalence (53.42%).

### Impact of the COVID-19 Pandemic and Sensitivity Analyses

When stratified by survey era, the prevalence of comorbid depression was consistently higher during the COVID-19 period than before the pandemic across all elastography thresholds (Table 2). Among individuals with LSM >15 kPa, depression prevalence increased from 10.12% prepandemic to 16.35% during the pandemic, while severe depression (PHQ-9 ≥15) more than doubled.

**Table 2:** Weighted Prevalence of Depression and ACLD/CSPH in the US Population Pre–COVID-19 and During COVID-19 Pandemic With Sensitivity Analyses

Outcome	Pre COVID-19 (2017–March 2020)				During COVID-19 (August 2021–August 2023)			
	ACLD% (95% CI)			CSPH^a^ % (95% CI)	ACLD % (95% CI)			CSPH^a^ % (95% CI)
	LSM 10–15 kPa^b^	LSM >15 kPa^c^	LSM >12 kPa^d^		LSM 10–15 kPa^b^	LSM >15 kPa^c^	LSM >12 kPa^d^	
Comorbid depression (PHQ-9 ≥10)	8.21 (4.61, 11.80)	10.12 (5.46, 14.79)	9.16 (5.68, 12.65)	10.61 (3.86, 17.36)	11.14 (4.91, 17.38)	16.35 (7.91, 24.79)	13.14 (8.36, 17.92)	13.63 (3.53, 23.74)
Severe depression (PHQ-9 ≥15)	3.45 (0.96, 5.94)	4.39 (1.12, 7.65)	3.48 (1.23, 5.74)	2.70 (0.00, 5.91)	4.18 (1.18, 7.18)	9.34 (3.57, 15.11)	6.87 (3.36, 10.38)	7.82 (1.56, 14.09)
Depression category (based on PHQ-9)								
No/minimal (0–4)	75.31 (68.19, 82.43)	67.08 (51.62, 82.54)	71.00 (61.24, 80.76)	61.54 (32.44, 90.64)	60.44 (54.98, 65.89)	61.14 (52.15, 70.13)	60.58 (53.33, 67.82)	68.77 (60.94, 76.60)
Mild (5–9)	16.29 (9.72, 22.86)	22.62 (6.90, 38.34)	19.70 (10.06, 29.34)	27.85 (0.00, 56.06)	26.52 (18.72, 34.32)	20.41 (13.82, 26.99)	24.23 (17.51, 30.95)	16.60 (7.02, 26.18)
Moderate (10–14)	4.87 (1.99, 7.75)	5.84 (1.55, 10.13)	5.76 (2.45, 9.08)	7.91 (2.72, 13.11)	8.15 (2.30, 14.01)	7.91 (3.06, 12.75)	7.25 (3.34, 11.17)	6.23 (0.00, 13.72)
Moderate to severe (15–19)	1.30 (0.00, 2.96)	3.35 (0.32, 6.38)	2.58 (0.52, 4.64)	2.70 (0.00, 5.91)	3.54 (0.35, 6.72)	8.22 (2.92, 13.53)	6.16 (3.17, 9.14)	6.60 (1.65, 11.55)
Severe (20–27)	2.23 (0.11, 4.34)	1.12 (0.00, 3.22)	0.96 (0.00, 2.27)	0 (0)	1.35 (0.00, 2.98)	2.32 (0.02, 4.62)	1.78 (0.01, 3.55)	1.79 (0.00, 4.72)
Sensitivity analyses								
Depression (PHQ-9 ≥10) PHQ-9 mean impute (≥7 items present)^e^	8.21 (4.61, 11.80)	10.12 (5.46, 14.79)	9.16 (5.68, 12.65)	10.61 (3.86, 17.36)	11.14 (4.91, 17.38)	16.35 (7.91, 24.79)	13.14 (8.36, 17.92)	13.63 (3.53, 23.74)
Depression (PHQ-9 ≥10) Missing PHQ-9 treated as 0^f^	8.21 (4.61, 11.80)	10.12 (5.46, 14.79)	9.16 (5.68, 12.65)	10.61 (3.86, 17.36)	11.14 (4.91, 17.38)	16.35 (7.91, 24.79)	13.14 (8.36, 17.92)	13.63 (3.53, 23.74)

a CSPH defined as LSM 15 to 20 kPa with PLT <110 G/L, or LSM >20 kPa and ≤25 kPa with PLT <150 G/L, or LSM >25 kPa regardless of platelet count.

b LSM 10 to 15 kPa is defined as “suggestive” of ACLD.

c LSM >15 kPa is defined as “highly suggestive” of ACLD.

d LSM >12 kPa was used as a single threshold definition of ACLD.

e Keeping all PHQ-9s, with ≥7 present, then missing 2 is mean of those present.

f Keeping all missing PHQ-9 as 0.

Depression severity distributions similarly shifted toward a higher symptom burden during the pandemic, with reductions in the proportion of individuals reporting no or minimal symptoms and corresponding increases in moderate-to-severe categories.

Sensitivity analyses using alternative PHQ-9 scoring strategies, including mean imputation for participants with ≥7 completed items and conservative assignment of missing responses as zero, yielded nearly identical prevalence estimates, confirming the robustness of the primary findings.

### Factors Associated With Comorbid Depression in Advanced Chronic Liver Disease

In multivariable logistic regression analyses to assess the risk factors associated with comorbid depression in individuals with ACLD (Table 3), female sex was strongly associated with higher odds of depression (aOR 3.51; 95% CI 1.98, 6.23; *P* < .001). Greater sedentary time was independently associated with depression (aOR 1.012 per hour/day; 95% CI 1.006, 1.018; *P* = .021), whereas older age was inversely associated (aOR 0.97 per year; 95% CI 0.94, 0.99 *P* = .025).

**Table 3:** Risk Factors Associated With Depression in ACLD in the US Population

Effect	ACLD			
	Adjusted odds ratio^a^	95% confidence limits		*P* value
Age	0.965	0.936	0.995	.0245
Gender				
Female vs male	3.512	1.981	6.227	<.0001
Race				
Mexican American vs NH White	1.787	0.552	5.784	.3236
Other Hispanic vs NH White	2.911	0.905	9.361	.0719
NH Black vs NH White	0.267	0.100	0.713	.0098
NH Asian vs NH White	1.314	0.192	9.013	.7754
Non-Hispanic other vs NH White	0.493	0.122	1.991	.3118
Marital status				
Widowed/divorced/separated vs married/living with partner	1.92	0.31	11.76	.4728
Never married vs married/living with partner	1.03	0.19	5.54	.9724
Education status				
High school or equivalent vs less than high school	1.40	0.40	4.97	.5905
Above high school vs less than high school	0.29	0.11	0.80	.0157
Smoking status				
Current smoker vs nonsmoker	1.479	0.600	3.646	.3850
Past smoker vs nonsmoker	1.170	0.393	3.480	.7721
Body mass index, kgm2	1.012	0.972	1.053	.5483
Sedentary activity, hours/day	1.012	1.006	1.018	.0211
Income/poverty ratio	0.852	0.704	1.032	.0994
Etiology of liver disease				
MASLD vs other	1.368	0.490	3.821	.5405
MetALD vs other	0.709	0.101	4.990	.7232
Excessive alcohol consumption vs other	6.556	1.442	29.818	.0163
Viral hepatitis (B and C) vs other	1.756	0.096	32.278	.6976
Postpandemic vs prepandemic	1.775	1.043	3.020	.0352
Known self-reported liver condition ever				
Yes vs No	2.339	0.843	6.493	.1002

MetALD, metabolic dysfunction and alcohol-associated liver disease; NH, non-Hispanic.

a Model was adjusted for age, race, sex, marital status, level of education, liver disease etiology, smoking, sedentary activity, income/poverty ratio, diabetes, hyperlipidemia and hypertension, known diagnosis of liver disease and era.

Compared to non-Hispanic White adults, non-Hispanic Black adults had significantly lower odds of depression (aOR 0.27; 95% CI 0.10, 0.71; *P* = .010). Higher educational attainment was protective, with adults above high school education exhibiting lower odds of depression than those with less than high school education (aOR 0.29; 95% CI 0.11, 0.80; *P* = .016).

Alcohol-associated liver disease, defined using NHANES alcohol-use questionnaire data based on sex-specific thresholds for excessive alcohol consumption, was independently associated with higher odds of depression (aOR 6.56, 95% CI 1.44, 29.82; *P* = .016). Depression was significantly more likely during the postpandemic period than during the prepandemic era (aOR 1.78; 95% CI 1.04, 3.02; *P* = .035).

BMI, smoking status, income, and remaining liver disease etiologies were not independently associated with depression after multivariate adjustment.

## Discussion

In this cross-sectional study of adults in the United States with ACLD and CSPH, we found that depression was a common comorbidity in this population. This finding aligns with existing literature, which shows that depression is prevalent among individuals with CLD.^8^ Depression negatively affects treatment adherence, disease progression, health-related quality of life, and long-term outcomes.^9^ In our cohort, the prevalence of depression was 14.6% among patients with ACLD and 15.1% among those with CSPH, which is lower than the previously reported prevalence of 29.2%–71.6% in patients with CLD.^9^ Differences in diagnostic criteria, study populations, and disease severity across studies likely contributed to this variability.

Our findings support a link between depression and diminished health-related quality of life in CLD. Previous studies have consistently indicated that individuals with CLD experience higher rates of depression than the general population.^10^ Notably, our work diverges from that of Khan et al,^9^ who reported no significant association between depression and age or sex in CLD. Differences in the underlying liver disease population may partly explain these findings. In contrast to our nationally representative NHANES cohort, which included a broader etiologic distribution with MASLD, metabolic dysfunction and alcohol-associated liver disease, excessive alcohol consumption, viral hepatitis, and other causes, the study by Khan et al was a single-center cross-sectional study from Abbottabad, Pakistan. Their paper reports that 86 of 111 participants had chronic hepatitis and that, among 86 patients with chronic viral hepatitis, 75 had hepatitis C. Their study also excluded patients with substance abuse except alcohol and did not report the prevalence of alcohol use disorder, other substance use disorders, or socioeconomic measures, limiting direct comparison with our cohort. While they found disease duration to be a key predictor of depressive symptoms, our data showed that younger adults with ACLD have disproportionately higher rates of depression. This aligns with broader mental health trends in the general population, where depression is more prevalent among younger individuals. This is consistent with the findings of Jiang et al,^11^ who observed elevated depression rates among young adults during the COVID-19 pandemic due to heightened isolation and economic pressures.

We also found that female sex was associated with higher odds of depression, supporting the work by Shafi et al,^12^ and reflecting well-established epidemiologic patterns in the general population. This sex disparity may be due to several interacting factors, including sex-related differences in stress-response and inflammatory pathways, the effects of hormonal fluctuations across the reproductive lifespan, and psychosocial burdens such as caregiving demands, trauma exposure, and socioeconomic stressors, all of which may increase vulnerability to depressive symptoms in women.^13^^,^^14^ Socioeconomic factors also had a significant impact. Poverty and unemployment were both associated with higher odds of depression among patients with ACLD, consistent with the findings in the general population. Psychological stressors, such as reduced functional capacity, economic hardship, mobility limitations, and loss of social roles may contribute to these outcomes.^15^ Moreover, the relationship appears bidirectional as depression can further impair functioning and reduce economic stability.

The racial and ethnic patterns in our study were consistent with national trends. Non-Hispanic Black patients had lower odds of depression than non-Hispanic White patients, aligning with broader US data showing higher rates of depression among White adults than among African American, Asian American, and Hispanic populations.^16^ Although research on racial differences in depression within CLD populations is limited, 1 study found that Hispanic ethnicity was inversely associated with depression in cirrhosis,^10^ reinforcing the need to consider sociocultural factors in mental health screening, even in patients with ACLD.

Patients with alcohol-related chronic liver disease demonstrated significantly higher odds of depression, consistent with previous findings showing increased depressive burden in alcohol-related liver disease, particularly in the pretransplant setting.^12^^,^^17^ In this study, excessive alcohol consumption was defined based on intake thresholds rather than formal diagnostic criteria for alcohol use disorder, which may capture a broader population at risk. Strikingly, lifetime depression has been identified as a strong predictor of mortality in patients with alcohol-related liver disease, doubling the risk of death.^18^ This finding highlights the importance of early psychiatric assessment in this subgroup.

Finally, our study captured the broader mental health impact of the COVID-19 pandemic, which led to increased depression rates across the general population.^19^ Similar patterns were observed among individuals with ACLD, which were likely driven by prolonged isolation, reduced social support, economic instability, and disruptions in medical care. Pandemic-related increases in alcohol use and alcohol-related harm may also have contributed to the higher depression prevalence observed during the COVID-19 period. In our cohort, excessive alcohol consumption was associated with higher depression burden, and prior literature has shown that the pandemic was accompanied by increased alcohol use, alcohol-related complications, and alcohol-associated liver disease mortality, suggesting potential residual confounding that was not directly modeled in our analysis.^20^^,^^21^ These findings highlight the need for integrated mental health support within the hepatology care pathways.

### Strengths and Limitations

The key strengths of this study include the use of nationally representative data, objective elastography-based liver disease definitions, and multiple validated approaches to PHQ-9 scoring, with sensitivity analyses demonstrating robust findings across assumptions regarding missing data.

This study has several limitations that merit consideration. The cross-sectional design precludes causal inference and limits our ability to determine the directionality between depression, alcohol use, and sedentary behavior. Depression was assessed using the PHQ-9 rather than clinical diagnosis, although this instrument has been well validated for epidemiologic research. The sample sizes within certain CSPH subgroups were modest, resulting in wider CIs. In addition, other substance use disorders were not evaluated because harmonized substance-use measures were not consistently available across the included NHANES cycles; therefore, residual confounding related to nonalcohol substance use may remain. This limitation is particularly relevant for participants with viral hepatitis, among whom opioid use disorder may be an important unmeasured comorbidity and potential confounder of depression risk. Finally, the viral hepatitis status in later NHANES cycles relied on self-reporting, which may have introduced misclassification.

## Conclusion

Depression is a significant and underrecognized comorbidity among adults with ACLD and CSPH in the United States. Younger age, female sex, alcohol-related liver disease, socioeconomic disadvantage, and post–COVID-19 period were all associated with higher odds of depression. Racial and ethnic differences observed in the general population were reflected in this cohort, with non-Hispanic Black patients showing lower odds of depression than non-Hispanic Whites. These findings underscore the need for routine mental health screening, as well as structured substance use screening and treatment, particularly for alcohol use disorder, within hepatology practice, with integrated care pathways that address both psychiatric and addiction-related comorbidity.

Incorporating structured psychiatric assessment into hepatology practice may improve the overall quality of life, optimize disease management, and potentially reduce mortality in this vulnerable population.
